# Ascorbic Acid Enhances the Inhibitory Effect of Theasaponins against *Candida albicans*

**DOI:** 10.3390/ijms251910661

**Published:** 2024-10-03

**Authors:** Yuhong Chen, Ying Gao, Junfeng Yin

**Affiliations:** 1 Key Laboratory of Tea Biology and Resources Utilization, Tea Research Institute, Chinese Academy of Agricultural Sciences, Ministry of Agriculture, 9 South Meiling Road, Hangzhou 310008, China; chenyuhong@tricaas.com; 2 Tea Research Institute, Hunan Academy of Agricultural Sciences, Changsha 410125, China

**Keywords:** *Candida albicans*, vitamin C (ascorbic acid), theasaponins, enhancement

## Abstract

*Candida albicans* (*C. albicans*) is a main cause of hospital-acquired fungal infections. Combination therapy is promising as a novel anti-*C. albicans* strategy because of its better efficacy. Theasaponins are pentacyclic triterpenes in the *Camellia* genus with multiple biological activities. Our previous studies prove that theasaponins display inhibitory activity against *C. albicans*. Ascorbic acid (VC) is a vitamin found in many plants that shows potential in combination therapy. However, whether VC enhances the activity of theasaponins remains unclear. In this study, the checkerboard micro-dilution method was used to assess the effect of VC (0–80 mmol/L) on the anti-*C. albicans* effect of theasaponins (0–1000 μg/mL). Then, the effects of theasaponins (31.25 μg/mL), VC (80 mmol/L), and theasaponins (31.25 μg/mL) + VC (80 mmol/L) on *C. albicans* planktonic cells and different stages of biofilm formation were assessed. Transcriptomic analysis was conducted to investigate the molecular mechanisms. According to the results, VC enhanced the anti-planktonic and anti-biofilm effect of theasaponins against *C. albicans*. The minimum inhibitory concentration of theasaponins was significantly decreased and the fungicidal efficiency was increased with the addition of VC. VC remarkably aggravated the suppression of theasaponins with regard to various virulence factors of *C. albicans*, including adhesion, early biofilm formation, mature biofilm, cell surface hydrophobicity, and phospholipase activity. Compared with the theasaponins or VC groups, the level of intracellular reactive oxygen species was higher, while the levels of mitochondrial membrane potential and adenosine triphosphate were lower in the combination group, suggesting more severe oxidative stress, mitochondrial injury, and energy deficiency. Transcriptomic analysis revealed that the combination predominantly suppressed the pathways of glycolysis, glycerophospholipid metabolism, glutathione metabolism, and cysteine and methionine metabolism. This implied that energy deficiency and redox imbalance were associated with the anti-*C. albicans* activity of the combination. These results prove that VC enhances the inhibitory effect of theasaponins against *C. albicans* and that the combination has the potential to be used as a topical antifungal therapy or disinfectant.

## 1. Introduction

Fungal infections are a major cause of infectious disease-associated mortality around the world [1]. *Candida albicans* (*C. albicans*) is one of the leading causative agents of systemic fungal infections [2]. Azoles and polyenes are first-line anticandidal medications, but their efficacy is under threat. More and more *C. albicans* strains acquire resistance to them, and the spread of these resistant strains poses clinical challenges [3,4]. The application of combinational agents is one of the most promising strategies to tackle the situation. The combinational agents consist of two antifungal agents or one antifungal agent with an adjunct agent [5]. Compared with the use of a single drug, the application of combinational agents causes greater stress for the survival of fungal cells by targeting multiple sites, resulting in enhanced efficacy and retarded evolution of resistance [6]. Meanwhile, the synergy between combinational agents lowers the dosage of each agent, thereby decreasing toxicity and side effects [7]. At present, there is some research on the combination treatment of *C. albicans*. The combination of anidulafungin and amphotericin B displayed stronger activity than individual agents in *Candida* biofilm inhibition in vitro [8]. The combination of fluconazole and ginkgolide B (a terpene lactone from the *Ginkgo biloba* leaf) had a synergistic effect against *C. albicans* in planktonic and biofilm states [7].

Natural compounds are a source of antifungal agents. Theasaponins are pentacyclic triterpenes in the *Camellia* genus, specifically accumulated in seeds and flowers. The content of theasaponins in *Camellia sinensis* seeds ranges from 16.93% to 31.82%, depending on the cultivar [9]. The structure of a typical theasaponin is composed of triterpenoid aglycone/sapogenin, sugar moiety, and organic acid moiety. About 70 types of theasaponins have been identified. Among them, theasaponin E1, assamsaponin G, and assamsaponin A are the most abundant ones, accounting for 28.2%, 13.4%, and 9.7% of the total theasaponins, respectively [9]. Theasaponins are natural non-ionic surfactants and display inhibitory activity against diverse fungal pathogens, such as the plant pathogen *Valsa mali* [10] and the dermatophyte *Trichophyton rubrum* [11]. Our previous study confirms the effectiveness of theasaponins on the inhibition of the conditional pathogen *C. albicans* [12]. However, the working concentration range is close to the toxic concentration range [13]. It is necessary to increase the safety without hampering the efficacy. The use of combinational agents is a possible approach.

Ascorbic acid, also known as vitamin C (VC), is a water-soluble compound found in fruits, vegetables, and medicinal plants. It is essential for the proper functioning of animals and humans. It exhibits excellent antioxidant capacity and acts as a cofactor for many enzymes, participating in normal metabolic processes and homeostasis, such as iron absorption, epigenetic modifications, and collagen synthesis [14]. Several studies indicate the potential of VC in combination with therapeutic agents. Shivavedi et al. [15] proved that a metformin and VC combination ameliorated type 2 diabetes and comorbid depression in rats. High-dose VC had synergy with many standard chemotherapies and mitigated the side effects [16]. Antimalarials were prescribed with VC or other antioxidants to attenuate malaria infection-induced oxidative stress on the host [17]. The outcomes of VC with anticandidal agents vary. VC improved the efficacy and decreased the cytotoxicity of amphotericin B by maintaining its molecular stability [18,19]. Conversely, it impaired the susceptibility of *C. albicans* to ketoconazole, probably because the antioxidant activity of VC compromised the ketoconazole-induced accumulation of reactive oxygen species (ROS) [20].

Currently, the impact of VC on the anti-*C. albicans* activity of theasaponins remains unclear. In this study, the checkerboard micro-dilution method was used to assess the impact of VC on the susceptibility of *C. albicans* (ATCC 10231, a fluconazole-resistant strain) to theasaponins and screened out the proper combination. The effects of the combination and individual agent on the planktonic and biofilm *C. albicans* cells were evaluated and compared. Transcriptomic analysis was conducted to explore the underlying mechanisms. The results bring a new insight into the application of the combinational strategy to suppress *C. albicans*.

## 2. Results and Discussion

### 2.1. VC Increases the Susceptibility of Planktonic C. albicans to Theasaponins

According to the combination susceptibility test (Table 1), the minimum inhibitory concentration (MIC) of theasaponins against planktonic *C. albicans* was 125 μg/mL, which was consistent with our previous study [12]. VC itself could not inhibit *C. albicans* at the test concentrations, but it significantly increased the susceptibility of *C. albicans* to theasaponins. When the concentrations of VC ranged from 10 to 40 mmol/L (mM), the MIC of theasaponins dropped to 62.5 μg/mL. When the concentration of VC reached 80 mM, the MIC of theasaponins further dropped to 31.25 μg/mL. The minimum fungicidal concentration (MFC) of theasaponins against planktonic *C. albicans* was 125 μg/mL. When the concentrations of VC ranged from 40 to 80 mM, the MFC of theasaponins dropped to 62.5 μg/mL.

**Figure 1:**
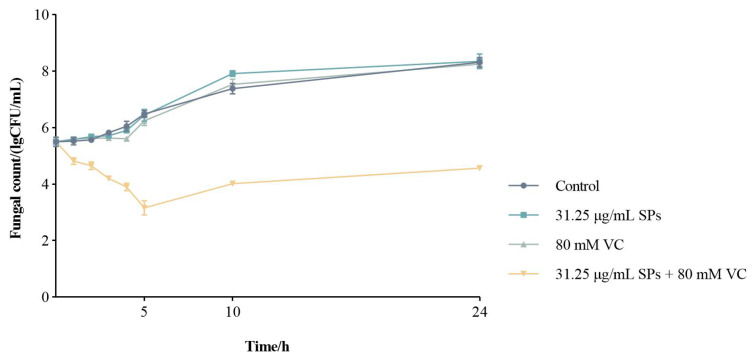
Time–kill curves of theasaponins (SPs) and ascorbic acid (VC).

The time–kill curves (Figure 1) showed that 80 mM VC and 31.25 μg/mL theasaponins individually had little inhibitory activity against the growth of planktonic *C. albicans* cells. Their combination exhibited significantly enhanced inhibitory activity. The cell numbers of the combination group continued decreasing during the initial 5 h, slightly increased at 10 h, and remained steady ever after. After 24 h of incubation, the cell number of the combination group was only half of that of the other three groups.

The results proved that the addition of VC remarkably enhanced the antifungal efficiency of theasaponins.

### 2.2. VC Enhances the Anti-Biofilm Effect of Theasaponins against C. albicans

As a dimorphic fungus, the ability to switch morphology and form biofilms is vital to the pathogenesis of *C. albicans* [21]. The biofilm development usually requires 24–48 h and includes a series of steps, such as adhesion, maturation, and dispersal [21]. At first, the yeast cells adhere to the substrate and form a basal cell layer. Then, the cells undergo proliferation and filamentation into hyphae. Later, the cells are embedded in the extracellular polymeric substance matrix as the biofilm matures. Finally, non-adherent yeast cells are released from the mature biofilm to initiate new biofilm formation or invade host tissues. Compared with planktonic cells, biofilm cells are more resistant to antifungal drugs, such as amphotericin B and fluconazole [22]. Our previous study revealed that three theasaponin monomers, which were theasaponin E1, theasaponin E2, and assamsaponin A, dose-dependently suppressed the adhesion, biofilm formation, and mature biofilm against *C. albicans* [23]. As with other antifungal drugs, the biofilm eradication concentration of theasaponin monomers was much higher than the biofilm formation inhibitory concentration. Rege et al. [24] found that incubation with VC for 48 h prevented the biofilm formation of *C. albicans* by 75%. These findings showed that both theasaponins and VC had anti-biofilm activity. In this study, the effect of the theasaponins and VC combination on different stages of biofilm formation was investigated.

**Figure 2:**
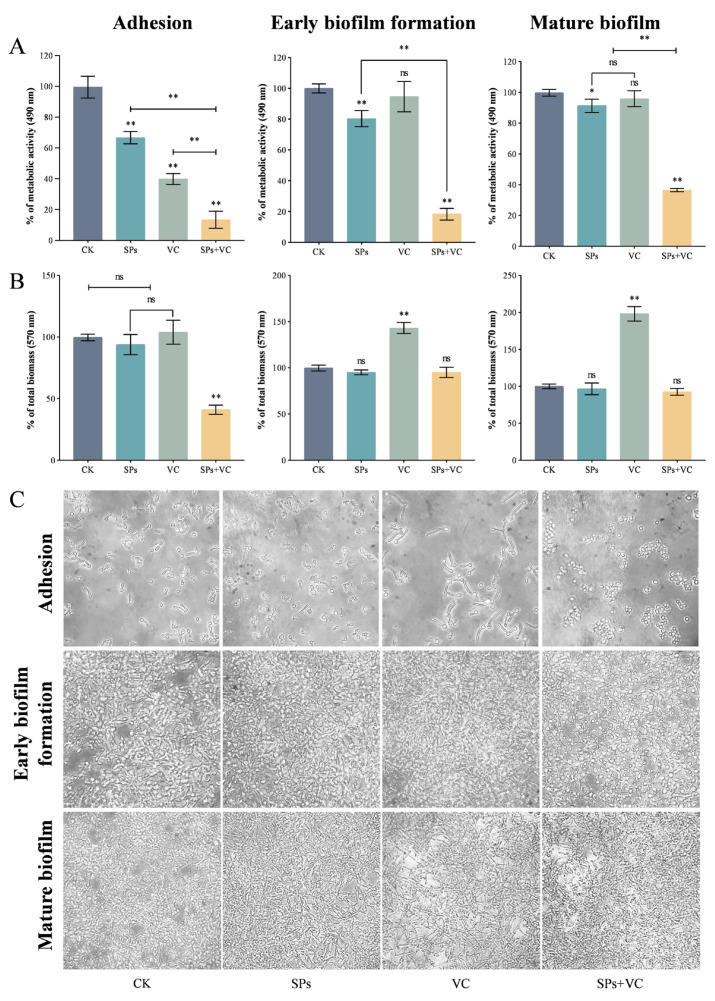
SPs and VC inhibited the adhesion, early biofilm formation, and mature biofilm of *C. albicans*. (**A**) Quantitative analysis of the XTT reduction assay; (**B**) quantitative analysis of the crystal violet staining assay; (**C**) microscopic observations (100×). The data represent the average (±standard deviation, SD) of six independent experiments. To assess the effect on the adhesion, *C. albicans* cells were co-incubated with vehicle or agent(s) for 1.5 h. To assess the effect on the early biofilm formation, *C. albicans* cells were incubated in the well plate for 1.5 h, and then treated with vehicle or agent(s) for 24 h. To assess the effect on the mature biofilm, *C. albicans* cells were incubated in the well plate for 24 h, and then treated with vehicle or agent(s) for 24 h. “**” above the column indicates significant differences (*p* < 0.01); “*” indicates significant differences (*p* < 0.05); and “ns” indicates insignificant differences (*p* ≥ 0.05). CK is short for the control group; SPs is short for the theasaponins group; VC is short for the ascorbic acid group; and SPs + VC is short for the combination group.

At the initial stage of biofilm formation (i.e., the adhesion stage), the VC and theasaponins individually significantly inhibited the metabolic activity of *C. albicans* cells, according to the results of the XTT reduction assay (Figure 2A). Their combination showed a stronger effect, reducing the metabolic activity of *C. albicans* cells by 90%. VC and theasaponins individually had no inhibition effect on the biomass of *C. albicans* cells, according to the results of the crystal violet staining assay, but their combination did (Figure 2B). Microscopic observation revealed that the morphology of each group varied (Figure 2C). In the control or theasaponins group, most of the cells grew either as a budding yeast or in the filamentous pseudohyphal form. In the VC group, the majority of cells were in the filamentous pseudohyphal form and were much longer than those in the control or theasaponins group, indicating that VC significantly promoted the elongation of pseudohyphae. It was possible that VC impacted the septum formation or microtubules. Further experiments, such as fluorescence confocal microscopy examination and electron microscopy examination, are needed to figure it out. Conversely, the cells in the combination group remained as yeast. These results suggested that the combination of VC and theasaponins displayed stronger inhibitory activity by disrupting the morphological transition.

At the early stage of biofilm formation, the theasaponins alone reduced the metabolic activity of *C. albicans* cells, while the VC could not (Figure 2A). The combination showed a synergy in decreasing the metabolic activity. However, none of the treatments were able to decrease the biomass (Figure 2B). The morphology of the cells in the VC or theasaponins group was similar to that in the control group and was mostly in the hyphal form (Figure 2C). The cells in the combination group were in the unicellular or budding yeast form, suggesting the potential role of the combination in early biofilm formation through the disruption of hyphal development.

For mature biofilm, the theasaponins alone slightly lowered the metabolic activity of *C. albicans* cells. When combined with VC, the inhibition was dramatically enhanced (Figure 2A). Likewise, none of the treatments decreased the biomass (Figure 2B). Microscopic examination showed that the structure of biofilm in the combination group was less confluent than the other three groups (Figure 2C).

The above results demonstrated that the combination of VC and theasaponins effectively inhibited the adhesion, morphological transition, hyphal development, and mature biofilm of *C. albicans*. The inhibition was attenuated along with the maturity of the biofilm. The saponins mainly displayed anti-biofilm activity by reducing the metabolic activity rather than the biomass. The VC augmented the anti-metabolic activity of the theasaponins. Despite the fact that VC promoted the elongation of pseudohyphae by itself, when combined with theasaponins they cooperated to reduce the proportion of elongated cells. The underlying mechanism still remains mysterious.

### 2.3. VC Aggravates Theasaponins-Induced Reduction in Cell Surface Hydrophobicity (CSH) and Extracellular Phospholipase

**Figure 3:**
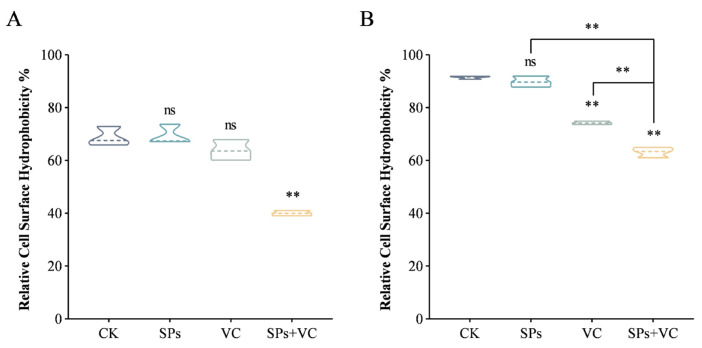
Effect of SPs and VC on the cell surface hydrophobicity of *C. albicans* at the adhesion stage (**A**) and mature biofilm (**B**). “**” above the column indicates significant differences (*p* < 0.01), and “ns” indicates insignificant differences (*p* ≥ 0.05).

In addition to anti-biofilm activity, the effects of the combination on the virulence factors of *C. albicans* were also evaluated by measuring the CSH and extracellular phospholipase. CSH refers to a cell’s affinity for a hydrophobic versus hydrophilic environment, which impacts virulence and biofilm formation [25]. Cells with higher CSH prefer a hydrophobic environment. They adhere more readily to host tissue, possess higher biofilm formation ability, and are more resistant to phagocytic killing, displaying stronger pathogenicity [26]. Extracellular polysaccharides and components on the surface of the cell wall, including mannoproteins, glucans, lipids, and chitin, are responsible for the CSH of *C. albicans* [25]. According to the results, the CSH of *C. albicans* at the adhesion stage was much lower compared with that in mature biofilm (Figure 3A). At the adhesion stage, only the combination significantly decreased the CSH by 40%. It was consistent with the microscopic observation that most of the cells in the combination group remained in the yeast form (Figure 2C), implying that the combination suppressed the adhesion by reducing CSH. For mature biofilm (Figure 3B), VC alone decreased the CSH by 19%, and the combination decreased the CSH by 31%, suggesting that the combination was more capable than the individual agent.

Extracellular phospholipase is one of the hydrolytic enzymes secreted during the pathogenesis of *C. albicans* to enhance survival in the host [27]. The activity of phospholipase was measured as the Pz value. A higher Pz value indicates a lower extracellular phospholipase activity. Exposure to antifungal drugs, such as nystatin, amphotericin B, caspofungin, and ketoconazole, significantly reduced the phospholipase activity of *C. albicans* [28]. At the adhesion stage, only the combination significantly increased the Pz value (Table 2). Likewise, for mature biofilm, only the combination significantly increased the Pz value. Combined with the results in Section 2.3 and the previous study [29], it was deduced that reduced metabolic activity and changes in cell surface structures might contribute to the decrease in phospholipase activity.

These findings indicated that the combination hampered the adhesion and invasion of *C. albicans* cells to weaken the pathogenicity by decreasing the CSH and extracellular phospholipase activity.

### 2.4. VC Exacerbates the Oxidative Stress and Energy Metabolism Decline in Theasaponins-Treated C. albicans Cells

**Figure 4:**
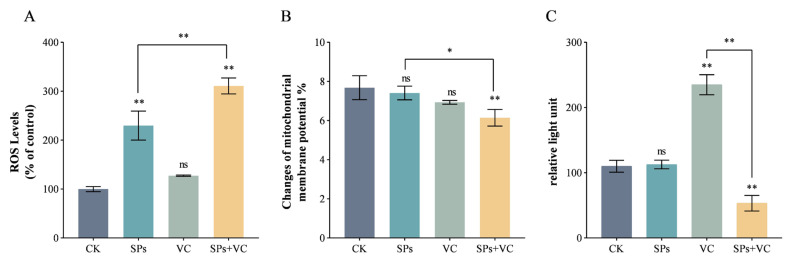
Effect of SPs and VC on (**A**) the intracellular reactive oxygen species (ROS), (**B**) mitochondrial membrane potential (MMP), and (**C**) intracellular adenosine triphosphate (ATP) of *C. albicans*. “**” above the column indicates significant differences (*p* < 0.01), “*” indicates significant differences (*p* < 0.05), and “ns” indicates insignificant differences (*p* ≥ 0.05).

The induction of intracellular oxidative stress is a pivotal molecular mechanism of many antifungal medications. ROS are key signaling molecules which participate in the process. At the test concentration, theasaponins alone significantly increased the intracellular ROS level, while VC alone had no impact (Figure 4A). Although VC was well known as an antioxidant, the intracellular ROS level in the combination group was much higher than that in the theasaponins group. Avci et al. [30] found that 90 mM of ascorbate killed *C. albicans* in a shaking culture, and the toxicity of ascorbate was attributed to the generation of hydroxyl radicals via a Fenton reaction. It was speculated that the combination of theasaponins and VC might also enhance the toxicity by inducing greater oxidative stress via the triggering of the Fenton reaction, with theasaponins increasing the substrate (e.g., H_2_O_2_) concentration and VC accelerating the regeneration of the catalyst (e.g., Fe^2+^).

The relationship between ROS and mitochondria is complex and bidirectional. Excessive ROS can result in mitochondrial damage [31]. And mitochondrial dysfunction leads to ROS generation. The mitochondrial membrane potential (MMP) is a reliable indicator of mitochondrial health [32]. It is generated by proton pumps and forms the transmembrane potential of hydrogen ions together with the proton gradient to control the mitochondrial capacity of adenosine triphosphate (ATP) generation via oxidative phosphorylation [33]. The level of MMP is relatively stable under normal conditions. A reduction in MMP is a hallmark of mitochondrial dysfunction, which relates to impaired bioenergetics and increased ROS generation [34]. At the test concentration, neither the theasaponins nor VC alone significantly decreased MMP (Figure 4B). It is noteworthy that the treatment of theasaponins alone induced intracellular ROS but did not reduce MMP, suggesting that the low concentration of theasaponins did not severely damage the mitochondria and that the extra ROS were probably not from the mitochondria. In any case, the combination of theasaponins and VC effectively reduced MMP. The MMP level of the combination group was 19.9% lower than that in the control group. It indicated that theasaponins and VC coordinated to disrupt mitochondrial function.

As mitochondria are the main powerhouses that produce ATP, the intracellular ATP level was monitored (Figure 4C). The theasaponins alone had no impact on the intracellular ATP level; the VC dramatically increased the intracellular ATP level, while the combination group had the lowest intracellular ATP level, which was 48.5% of that in the control group. A previous study revealed that VC enhanced the activity of the mitochondrial electron transport chain by mediating electrons from coenzyme Q to cytochrome c and increased ATP production under normal conditions [35,36], which was consistent with the current result. Under oxidative stress, the ascorbyl free radical was accumulated, and it impaired mitochondrial respiration, probably by causing an arrest of the electron flow between Complex III and Complex IV, resulting in decreased ATP production [37]. The hypothesis might explain the significant decrease in ATP production in the combination group. Another assumption was that the combination decreased ATP production in a mitochondria-independent way. Despite the fact that the majority of ATP is generated by mitochondria via oxidative phosphorylation, ATP is also synthesized in the cytosol via glycolysis.

### 2.5. The Combination of VC and Theasaponins Modulates the Transcriptomic Profile, Predominantly Targeting Energy Metabolism

**Figure 5:**
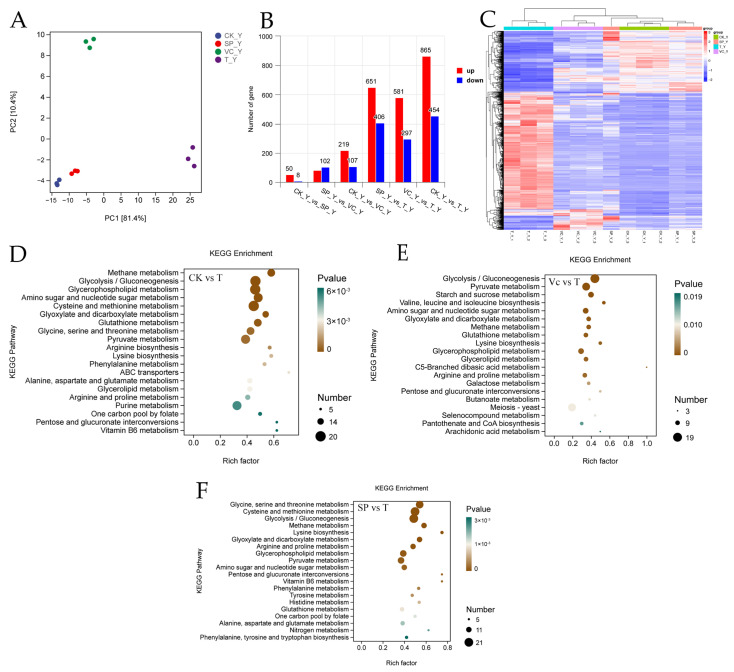
Effect of SPs and VC on the transcriptomic profile. (**A**) The principal component analysis plot based on differentially expressed genes (DEGs). (**B**) The numbers of DEGs between groups. (**C**) The hierarchical clustering analysis based on DEGs. (**D**–**F**) KEGG enrichment analysis of DEGs between (**D**) the control group and the SPs + VC combination group, (**E**) the VC group and the SPs + VC combination group, and (**F**) the SPs group and the SPs + VC combination group. CK_Y and CK in the figures are short for the control group; SP_Y and SP are short for the theasaponins group; VC_Y and VC are short for the ascorbic acid group; and T_Y and T are short for the combination group.

To unveil the underlying mechanisms, transcriptomic analysis by RNA sequencing was conducted. The Q20 of each piece of raw data was over 97%, indicating high accuracy and reliability. A principal component analysis plot based on the differentially expressed genes (DEGs) showed that the dots representing each group were isolated, suggesting that the transcriptomic profile of each group was distinguishable (Figure 5A). And the distance between the control group dots and the theasaponins group dots was the shortest, indicating the high similarity in the transcriptomic profile of the two groups. Compared with the control group, 58, 326, and 1319 DEGs were identified in the theasaponins group, VC group, and the theasaponins+VC group, respectively (Figure 5B). Compared with the theasaponins+VC group, 1057 and 878 DEGs were identified in the theasaponins group and VC group, respectively. The hierarchical clustering analysis also demonstrated that the transcriptomic profile of the combination group was more distinctive than that of the other three groups, followed by the VC group (Figure 5C).

**Figure 6:**
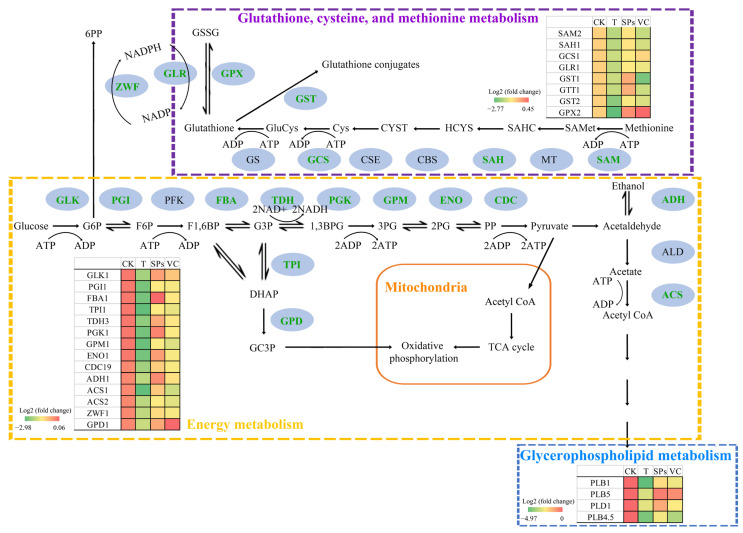
Heatmaps of DEGs encoding key enzymes in energy metabolism; glutathione, cysteine, and methionine metabolism; and glycerophospholipid metabolism. Gene name marked in green over the purple dot indicates that the gene was significantly downregulated in the theasaponins–VC combination group compared with the control group. Short names and corresponding full names are listed as follows. CK—the control group; SPs—the theasaponins group; VC—the ascorbic acid group; T—the combination group; G6P—glucose-6-phosphate; F6P—fructose-6-phosphate; F1,6BP—fructose-1,6-bisphosphate; G3P—glyceraldehyde-3-phosphate; 1,3BPG—1,3-bisphosphoglycerate; 3PG—3-phosphoglycerate; 2PG—2-phosphoglycerate; PP—phosphoenolpyruvate; DHAP—dihydroxyacetone phosphate; GC3P—glycerol-3-phosphate; Acetyl CoA—acetyl-coenzyme A; TCA cycle—tricarboxylic acid cycle; SAMet—S-adenosyl-methionine; SAHC—S-adenosyl-homocysteine; HCYS—homocysteine; CYST—cystathionine; Cys—cysteine; GluCys—γ-glutamylcysteine; GSSG—glutathione disulfide; ATP—adenosine triphosphate; ADP—adenosine diphosphate; NAD+—nicotinamide adenine dinucleotide; NADH—reduced nicotinamide adenine dinucleotide; NADPH—reduced nicotinamide adenine dinucleotide phosphate; NADP—nicotinamide adenine dinucleotide phosphate; 6PP—6-phosphogluconolacone; GLK—glucokinase; PGI—glucose-6-phosphate isomerase; PFK—phosphofructokinase; FBA—fructose-bisphosphate aldolase; TDH—glyceraldehyde-3-phosphate dehydrogenase; PGK—phosphoglycerate kinase; GPM—phosphoglycerate mutase; ENO—enolase; CDC—pyruvate kinase; TPI—triosephosphate isomerase; GPD—glycerol-3-phosphate dehydrogenase [NAD(+)]; ADH—alcohol dehydrogenase; ALD—acetaldehyde dehydrogenase; ACS—acetyl-coenzyme A synthetase; SAM—S-adenosylmethionine synthase; MT—methyltransferase; SAH—S-adenosylhomocysteinase; CBS—cystathionine β-synthase; CSE—cystathionine γ-lyase; GCS—γ-glutamylcysteine synthetase; GS—glutathione synthase; GST—glutathione S-transferase; GPX—glutathione peroxidase; GLR—glutathione reductase; ZWF—glucose-6-phosphate 1-dehydrogenase; PLB—phospholipase B; PLD—phospholipase D.

The Kyoto Encyclopedia of Genes and Genomes (KEGG) analysis (Figure 5D–F) revealed that many DEGs between the control group and the combination group were associated with glycolysis. Glycolysis is highly conserved among living organisms and occurs in the cytosol of the cell without the use of oxygen. It is the metabolic pathway that breaks down glucose into pyruvate and produces ATP and NADH [38]. Pyruvate can be oxidized to acetyl-CoA and enter the tricarboxylic acid cycle to produce energy in the presence of oxygen. Pyruvate can also serve as a key intermediate molecule in the metabolisms of proteins, fats, and carbohydrates [39]. Compared with the control group, the theasaponins or VC alone did not significantly downregulate the transcription of genes related to the glycolysis process (Figure 6). In contrast, the combination dramatically inhibited genes encoding glycolytic enzymes, including the rate-limiting enzymes glucokinase and pyruvate kinase. The results were in accordance with the results in Section 2.4 and implied that the combination might induce energy metabolism decline by targeting the glycolysis process to inhibit *C. albicans*.

It is noteworthy that the combination significantly inhibited glycerol-3-phosphate dehydrogenase [NAD(+)] (Figure 6), which catalyzes the formation of glycerol-3-phosphate in the cytosol by converting the glycolysis intermediate dihydroxyacetone phosphate and oxidizing nicotinamide adenine dinucleotide and is a key member in the glycerol-3-phosphate shuttle [40]. The shuttle delivers cytosolic reducing equivalents into mitochondrial oxidative phosphorylation [41]. The high flux through cytosolic glycerol-3-phosphate dehydrogenase is required to maintain redox balance. It has been reported that the shuttle is involved in the development and virulence of fungi [41,42]. The inhibition of the enzyme might exacerbate energy deficiency and oxidative stress.

In addition, pyruvate can undergo non-oxidative decarboxylation with the catalyzation of pyruvate decarboxylase to produce acetaldehyde. On one hand, acetaldehyde is converted by alcohol dehydrogenase 1 (ADH1) into ethanol at the end of glycolysis [43]. ADH1 locates on the cell wall surface and plays roles in biofilm formation, interactions between different species, and the development of drug resistance [43]. It is regarded as a potential target for the inhibiting of the fungal infection event [44]. The combination, but not theasaponins or VC alone, significantly decreased the transcription of ADH1 (Figure 6). It implied that the combination might lower the energy production and virulence of *C. albicans*. On the other hand, acetaldehyde is oxidized by acetaldehyde dehydrogenase to acetic acid; then, acetyl-CoA is generated by acetyl-coenzyme A synthetase (ACS). Acetyl-CoA is a central intermediate for energy metabolism and biosynthetic pathways [45]. The acs1-acs2 double mutant is not viable [46]. *C. albicans* strains depleted for ACS2 are unviable in the presence of most carbon sources, including glucose, acetate, and ethanol [47]. The combination remarkably suppressed the transcription of both ACS1 and ACS2 (Figure 6), which might decrease the viability of *C. albicans*.

Energy deficiency profoundly affects metabolisms, especially the energy-requiring ones. Among them, glutathione, cysteine, and methionine metabolisms are notable. Glutathione is a tripeptide consisting of L-glutamate, cysteine, and glycine [48]. It is a potent antioxidant which protects cells from oxidative damage and the toxicity of xenobiotics and maintains redox homeostasis [49]. Glutathione is synthesized by the sequential addition of cysteine to glutamate followed by the addition of glycine, through continuous two-step enzymatic reactions which depend on ATP [48]. The biosynthesis is determined by the availability of the sulfur amino acid precursor, cysteine, and the activity of the rate-limiting enzyme, γ-glutamylcysteine synthetase (GCS; also referred to as glutamate cysteine ligase) [50]. An important source of cysteine is the conversion of methionine via the transsulfuration pathway. With the catalyzation of S-adenosylmethionine synthase (SAM; also referred to as methionine adenosyltransferase), S-adenosyl-methionine (SAMet) is formed. Methyltransferase produces S-adenosyl-homocysteine (SAHC) from SAMet. S-adenosylhomocysteinase (also known as S-adenosyl-homocysteine hydrolase, SAH) catalyzes the reversible hydrolysis of SAHC to adenosine and homocysteine (HCYS). Cystathionine β-synthase (CBS) synthesizes cystathionine by the condensation of HCYS and serine. Thereafter, cystathionine is hydrolyzed by cystathionine γ-lyase (CSE) to generate cysteine [51]. The combination significantly suppressed the transcription of SAM and SAH (Figure 6), two enzymes in the biosynthetic pathway of cysteine, which reduced the availability of cysteine. Moreover, the combination effectively decreased the transcription of GCS, which suppressed the biosynthesis of glutathione. The combination inhibited the transcription of glutathione reductase (GLR), which blocked the regeneration of reduced glutathione from oxidized glutathione [52]. Interestingly, the combination also inhibited glutathione S-transferases, which at least partially disabled the detoxification function of glutathione. The lack and inactivation of glutathione might lead to the overaccumulation of intracellular ROS, which is in line with the result shown in Figure 4B, and finally injure the cells.

Additionally, there were DEGs annotated to the glycerophospholipid metabolism pathway, including several genes encoding lysophospholipase and phospholipases, such as phospholipase B1 (PLB1), B5 (PLB5), and D1 (PLD1). *C. albicans* contains five putative phospholipase B (PLB) genes, each of which encodes a PLB enzyme. PLB enzymes hydrolyze acyl ester bonds in phospholipids and lysophospholipids and catalyze lysophospholipase-transacylase reactions [53]. PLB enzymes are unnecessary for the normal growth and morphology of *C. albicans*, but they are upregulated in conditions favoring filamentous growth, suggesting their role in candidal virulence [54]. PLB1 is directly responsible for the pathogenicity of *C. albicans* and participates in the early steps of host invasion [55]. PLD enzymes hydrolyze the terminal phosphodiester bond of membrane phospholipids to release phosphatidic acid and catalyze a unique transphosphatidylation reaction using primary alcohols as nucleophilic acceptors [54]. PLD1 is constitutively expressed during yeast growth and stimulated during dimorphic transition [56]. The *Capld1* null mutants show attenuated virulence in candidiasis models [57]. This suggests that PLD1 is a potential virulence determinant due to its role as an important regulator of the yeast to hyphae transition in *C. albicans*. Theasaponins or VC alone did not significantly downregulate the transcription of phospholipases, but the combination did. The results were consistent with the results of the extracellular phospholipase activity (Table 2), implying that the combination decreased the virulence of *C. albicans* by targeting phospholipases.

Deducing from the above results, it was speculated that the inhibitory mechanisms of the combination were associated with the suppression of energy metabolism, the disability of glutathione, and the reduction in phospholipases.

Learning from previous references, there are many other possible anticandidal mechanisms worth attention. Pistoia et al. [58] found that all-trans retinoic acid induced plasma membrane damage in *C. albicans* cells and exhibited excellent activity on the growth and biofilm formation of *C. albicans*, indicating the plasma membrane as an important anticandidal target. Our previous study demonstrated that two theasaponin monomers increased the cell membrane permeability and disrupted the cell membrane integrity of *C. albicans* cells [12]. Further experiments are needed to investigate whether VC enhances the inhibitory activity of theasaponins by aggravating the plasma membrane damage of *C. albicans* cells. Sánchez-Fresneda et al. [59] revealed that *C. albicans* cells rapidly increased the endogenous synthesis of trehalose and D-arabitol under oxidative stress to counteract environmental challenges. Our current results indicated that theasaponins alone or combined with VC induced oxidative stress in *C. albicans* cells, but the effects on the synthesis of trehalose and D-arabitol were not investigated. In a future study, the concentrations of intracellular trehalose and other compatible solutes will be monitored to assess whether theasaponins and VC display anticandidal activity by hindering the synthesis of these defensive compounds.

## 3. Materials and Methods

### 3.1. Reagents

The *Candida albicans* strain (ATCC 10231) was purchased from Guangdong Microbial Culture Collection Center (Guangzhou, China). The VC was purchased from Shanghai Aladdin Biochemical Technology Co., Ltd. (Shanghai, China). The theasaponins were prepared according to our previously published method [12].

### 3.2. Combination Susceptibility Test

The checkerboard micro-dilution method was used to test the combination susceptibility of theasaponins and VC in the *Candida albicans* strain (ATCC 10231) [60]. Serial two-fold dilutions were prepared in broth for the theasaponins and VC, respectively. The final concentrations of theasaponins ranged from 0 to 1000 μg/mL; the final concentrations of VC ranged from 0 to 80 mM; and the final intensity of the *C. albicans* cells was 5 × 10^5^ CFU/mL. The cells were cultured at 30 °C for 24 h. MIC was set as the lowest concentration which inhibited the visible growth of *C. albicans*. For wells without visible growth of *C. albicans*, 10 μL of cell culture from each well was plated onto yeast extract–peptone–dextrose (YPD) agar and incubated overnight at 30 °C, respectively. MFC was defined as the lowest concentration where no colony growth was observed.

### 3.3. Time-Kill Curves

*C. albicans* cells from a single colony were cultured in YPD liquid medium and grown overnight at 30 °C in a shaking incubator at 200 rpm. The cell suspension was diluted to the intensity of 5 × 10^5^ CFU/mL and treated with vehicle (the control group), theasaponins (31.25 μg/mL), VC (80 mM or 14.1 mg/mL), and theasaponins (31.25 μg/mL) + VC (80 mM), respectively. The cells were grown at 30 °C in a shaking incubator at 200 rpm and sampled at 0 h, 1 h, 2 h, 3 h, 4 h, 5 h, 10 h, and 24 h. Ten microliters of each sample was serially diluted and spread onto YPD agar plates. After 24 h of incubation at 30 °C, the number of colonies was counted. The logarithm of the number of colonies counted (CFU)/mL is plotted versus time to construct the time–kill curve.

### 3.4. Determination of Adhesion, Early Biofilm Formation, and Mature Biofilm

To assess the adhesion, *C. albicans* cells were suspended in RPMI 1640 medium (2 × 10^6^ CFU/mL) and treated with vehicle, theasaponins (31.25 μg/mL), VC (80 mM), and theasaponins (31.25 μg/mL) + VC (80 mM), respectively. Two hundred microliters of each mixture was added into a 96-well plate. After incubating at 37 °C for 1.5 h, non-adherent cells were removed by washing the well with D-PBS twice.

To assess the early biofilm formation, a *C. albicans* cell suspension (2 × 10^6^ CFU/mL in RPMI 1640 medium, 100 μL/well) was added into a 96-well plate and incubated at 37 °C for 1.5 h. Non-adherent cells were removed by washing the well with D-PBS twice. The remaining cells were treated with vehicle (RPMI 1640 medium), theasaponins (31.25 μg/mL), VC (80 mM), and theasaponins (31.25 μg/mL) + VC (80 mM), respectively, at 37 °C for 24 h.

To assess the mature biofilm, a *C. albicans* cell suspension (2 × 10^6^ CFU/mL in RPMI 1640 medium, 100 μL/well) was added into a 96-well plate and incubated at 37 °C for 24 h. Non-adherent cells were removed by washing the well with D-PBS twice. The remaining cells were treated with vehicle (RPMI 1640 medium), theasaponins (31.25 μg/mL), VC (80 mM), and theasaponins (31.25 μg/mL) + VC (80 mM), respectively, at 37 °C for 24 h.

The effects of theasaponins, VC, and theasaponins+VC on the adhesion, early biofilm formation, and mature biofilm of *C. albicans* cells were evaluated in terms of metabolic activity and biomass.

The metabolic activity was determined using the 2,3-bis-(2-methoxy-4-nitro-5-sulphenyl)-(2H)-tetrazolium-5-carboxanilide (XTT) reduction assay. Adherent cells were stained with XTT solution at 37 °C for 1 h in the dark, and the absorbance at 490 nm was measured by a microplate reader (Synergy H1, BioTek Instruments, Inc., Winooski, VT, USA). The metabolic activity was calculated as follows:

$$Metabolic\;activity\;\left(\%\right)=\frac{A_{treatment}-A_{blank}}{A_{control}-A_{blank}}\times 100\%$$

The biomass was determined using the crystal violet staining assay [61]. Adherent cells were air-dried for 45 min, stained with 100 μL of 0.5% crystal violet solution for 15 min, washed with D-PBS to remove excess crystal violet, and then added to 100 μL of ethanol to release the dyes from the cells. The absorbance at 570 nm was measured by a microplate reader. The total biomass percentage was calculated as follows:

$$Total\;biomass\;\left(\%\right)=\frac{A_{treatment}-A_{blank}}{A_{control}-A_{blank}}\times 100\%$$

### 3.5. Determination of CSH

To determine the CSH of adherent cells, a *C. albicans* cell suspension (1 × 10^7^ CFU/mL in RPMI 1640 medium) was added into a 6-well plate and treated with vehicle (RPMI 1640 medium), theasaponins (31.25 μg/mL), VC (80 mM), and theasaponins (31.25 μg/mL) + VC (80 mM), respectively, for 90 min.

To determine the CSH of mature biofilm cells, a *C. albicans* cell suspension (1 × 10^7^ CFU/mL in RPMI 1640 medium) was added into a 6-well plate and incubated at 37 °C for 24 h. Then, the cells were treated with vehicle (RPMI 1640 medium), theasaponins (31.25 μg/mL), VC (80 mM), and theasaponins (31.25 μg/mL) + VC (80 mM), respectively, for 24 h.

After treatment, the cells were collected. Non-adherent cells were resuspended in the cell culture medium, and the suspension was transferred into a centrifuge tube. A cell scraper was used to scrape the adherent cells from the bottom of the well. The cells were suspended in the cell culture medium and transferred into the previous centrifuge tube. The merged cell suspension was centrifuged at 3000× *g* for 5 min, washed using D-PBS triple, and the suspension was diluted to an OD_600_ of 1.0. Then, 1.2 mL of cell suspension was mixed with 0.3 mL of n-octane, vortexed for 3 min, and kept still for 30 min. The optical density of the aqueous layer at 600 nm was measured using a microplate reader (Synergy H1, BioTek Instruments, Inc., Winooski, VT, USA). The CSH% was calculated as follows:

$$CSH\;\left(\%\right)=\frac{{OD}_{control}-{OD}_{treatment}}{{OD}_{control}}\times 100\%$$

where the *OD_control_* represents the *OD_600_* value of the cell suspension without the addition of n-octane.

### 3.6. Determination of Extracellular Phospholipase Activity

To determine the extracellular phospholipase activity of the adherent cells, a *C. albicans* cell suspension (1 × 10^5^ CFU/mL in RPMI 1640 medium) was mixed with vehicle (RPMI 1640 medium), theasaponins (31.25 μg/mL), VC (80 mM), and theasaponins (31.25 μg/mL) + VC (80 mM), respectively.

To determine the extracellular phospholipase activity of the mature biofilm cells, a *C. albicans* cell suspension (1 × 10^5^ CFU/mL in RPMI 1640 medium) was incubated at 37 °C for 24 h and then mixed with vehicle (RPMI 1640 medium), theasaponins (31.25 μg/mL), VC (80 mM), and theasaponins (31.25 μg/mL) + VC (80 mM), respectively.

One microliter of the cell suspension mixture was evenly distributed onto egg yolk emulsion agar and incubated at 37 °C for 4 days. The presence of sedimentation circles around the colonies indicated the production of phospholipase by the *C. albicans* strain. The diameter of the colony (d1) and the diameter of the precipitation zone (d2) were measured. The extracellular phospholipase activity was calculated as the ratio of d1/d2 and expressed as the Pz value. A higher Pz indicates a lower extracellular phospholipase activity. In detail, Pz = 1 indicates no activity, 0.90 < Pz ≤ 0.99 indicates extremely low activity, 0.80 < Pz ≤ 0.89 indicates low activity, 0.70 < Pz ≤ 0.79 indicates high activity, and Pz ≤ 0.69 indicates extremely high activity.

### 3.7. Determination of Intracellular ROS, MMP, and ATP Levels

A *C. albicans* cell suspension (1 × 10^7^ CFU/mL) was treated with vehicle, theasaponins (31.25 μg/mL), VC (80 mM), and theasaponins (31.25 μg/mL) + VC (80 mM), respectively, at 30 °C for 2 h and washed twice with PBS before tests. The intracellular ROS, MMP, and ATP levels were measured using corresponding commercial kits (Product Nos. S0033S, C2006, and S0026, Beyotime Biotech, Shanghai, China), respectively, according to the manufacturer’s instructions. The viability of the cells was measured using a cell counting kit-8 (Product No. C0037, Beyotime Biotech, Shanghai, China). The ROS level is presented as a percentage compared to the control group. The MMP level is expressed as the fluorescence intensity ratio of JC-1 monomer to aggregate.

### 3.8. Transcriptome Analysis

A *C. albicans* cell suspension (1 × 10^7^ CFU/mL) was treated with vehicle, theasaponins (31.25 μg/mL), VC (80 mM), and theasaponins (31.25 μg/mL) + VC (80 mM), respectively, at 30 °C for 2 h and washed twice with PBS.

Total RNA was extracted using a yeast RNA extraction kit (Aidlab Biotech, Beijing, China). The concentration and quality of the RNA were monitored. The cDNA libraries were constructed with an Illumina NovaSeq 6000 platform (Personalbio Technology, Shanghai, China) with paired-end reads. The clean reads were mapped to the *C. albicans* SC5314 genome sequence with HISAT2. DEGs between groups were screened out using the DESeq2 software (v 1.40.1), with |log2 fold change| > 1 and *p*-value < 0.05 as the criteria. Functional annotation was conducted based on the KEGG (http://www.genome.ad.jp/kegg/, accessed on 14 December 2023) databases.

### 3.9. Statistical Analysis

All the data are presented as mean ± standard deviation from at least three independent experiments. Statistical analysis was conducted with one-way ANOVA using SPSS (Version 22.0) to determine significant differences (*p* < 0.05, *p* < 0.01). GraphPad Prism (v9.4.1) was used to process the data and generate the figures.

## 4. Conclusions

VC not only sensitized the susceptibility of planktonic *C. albicans* to theasaponins, but also inhibited the adhesion, early biofilm formation, and mature biofilm of *C. albicans*. In addition, VC cooperated with theasaponins to attenuate the virulence of *C. albicans* by reducing the CSH and phospholipase activity. The combination significantly suppressed the transcription of genes encoding the enzymes involved in glycolysis and impaired mitochondrial function, both of which led to energy metabolism decline, with the decrease in intracellular ATP as a hallmark. Energy deficiency could further disrupt multiple fundamental biological processes, leading to cell damage and even death. The combination also elevated the intracellular ROS level, which was presumedly associated with the reduced glutathione metabolism. It should be mentioned that the VC concentration of the current combination is high, which limits the intravenous use. Novel drug delivery systems may be applied to solve the problem. Taken together, our findings demonstrated the enhancement of the anti-*C. albicans* activity of theasaponins by VC, providing evidence for the use of the theasaponins–VC combination as a topical antifungal therapy or disinfectant. More studies are needed to determine the detailed underlying molecular mechanisms.

## Figures and Tables

**Table 1:** The susceptibility of *Candida albicans* (*C. albicans*) ATCC 10231 to theasaponins (SPs) and ascorbic acid (VC).

		SPs (μg/mL)							
		0	15.625	31.25	62.5	125	250	500	1000
**VC (mM)**	0	+/NT	+/NT	+/NT	+/NT	-/-	-/-	-/-	-/-
	1.25	+/NT	+/NT	+/NT	+/NT	-/-	-/-	-/-	-/-
	2.5	+/NT	+/NT	+/NT	+/NT	-/-	-/-	-/-	-/-
	5	+/NT	+/NT	+/NT	+/NT	-/-	-/-	-/-	-/-
	10	+/NT	+/NT	+/NT	-/+	-/-	-/-	-/-	-/-
	20	+/NT	+/NT	+/NT	-/+	-/-	-/-	-/-	-/-
	40	+/NT	+/NT	+/NT	-/-	-/-	-/-	-/-	-/-
	80	+/NT	+/NT	-/+	-/-	-/-	-/-	-/-	-/-

Data presented in each cell indicate the result of the determination of minimum inhibitory concentration/minimum fungicidal concentration. “+” means visible growth of the fungus, while “-” means no visible growth of the fungus. “NT” means not tested.

**Table 2:** Effect of SPs and VC on the extracellular phospholipase activity of *C. albicans* at the adhesion stage and mature biofilm.

	Adhesion		Mature Biofilm	
	Pz Value	Phospholipase Activity	Pz Value	Phospholipase Activity
Control	0.60 ± 0.02	Very high	0.53 ± 0.04	Very high
SPs	0.62 ± 0.01	Very high	0.56 ± 0.07	Very high
VC	0.56 ± 0.04	Very high	0.51 ± 0.03	Very high
SPs + VC	0.78 ± 0.05 **	High	0.74 ± 0.07 **	High

“**” indicates significant differences compared with the control group (*p* < 0.01).

## Data Availability

The data presented in this study are available upon request from the corresponding author.

## References

[1] Lee Y., Puumala E., Robbins N., Cowen L.E. (2021). Antifungal drug resistance: Molecular mechanisms in candida albicans and beyond. Chem. Rev..

[2] Lee Y., Robbins N., Cowen L.E. (2023). Molecular mechanisms governing antifungal drug resistance. NPJ Antimicrob. Resist..

[3] He X., Kusuya Y., Hagiwara D., Toyotome T., Arai T., Bian C., Nagayama M., Shibata S., Watanabe A., Takahashi H. (2024). Genomic diversity of the pathogenic fungus aspergillus fumigatus in japan reveals the complex genomic basis of azole resistance. Commun. Biol..

[4] Gao J., Wang H., Li Z., Wong A.H., Wang Y.Z., Guo Y., Lin X., Zeng G., Liu H., Wang Y. (2018). Candida albicans gains azole resistance by altering sphingolipid composition. Nat. Commun..

[5] Scorzoni L., Fuchs B.B., Junqueira J.C., Mylonakis E. (2021). Current and promising pharmacotherapeutic options for candidiasis. Expert. Opin. Pharmacother..

[6] Spitzer M., Robbins N., Wright G.D. (2017). Combinatorial strategies for combating invasive fungal infections. Virulence.

[7] Li Y., Yang J., Li X., Su S., Chen X., Sun S., Li Y. (2020). The effect of ginkgolide b combined with fluconazole against drug-resistant candida albicans based on common resistance mechanisms. Int. J. Antimicrob. Agents.

[8] Reginatto P., Bergamo V.Z., Berlitz S.J., Guerreiro I.C.K., de Andrade S.F., Fuentefria A.M. (2020). Rational selection of antifungal drugs to propose a new formulation strategy to control candida biofilm formation on venous catheters. Braz. J. Microbiol..

[9] Chen Y.H., Gao Y., Han Z., Yin J.F. (2022). Analysis of the saponin contents and composition in tea seeds of different germplasms. J. Tea Sci..

[10] Li Z.Y., Sun Q., Ma N., Zhang F.J., Zhang S., Zhang Z.Q., Wang X.F., Sun P., You C.X., Zhang Z. (2023). Inhibitory effect of tea saponin on major apple-disease-inducing fungi. Phytopathology.

[11] Joshi R., Sood S., Dogra P., Mahendru M., Kumar D., Bhangalia S., Pal H.C., Kumar N., Bhushan S., Gulati A. (2013). In vitro cytotoxicity, antimicrobial, and metal-chelating activity of triterpene saponins from tea seed grown in kangra valley, india. Med. Chem. Res..

[12] Chen Y., Gao Y., Yuan M., Zheng Z., Yin J. (2023). Anti-candida albicans effects and mechanisms of theasaponin e1 and assamsaponin A. Int. J. Mol. Sci..

[13] Zhang M., Chen Z.Y., Tian D., Li Z.Q., Wang S.N., Huo Y.J., Song L., Lu J., Sheng J., Ji X. (2023). Effects of theasaponin e1 on the regulationglucose uptake of c2c12 myoblasts pi3k/akt/mtor signaling pathway. CyTA–J. Food.

[14] Dosedel M., Jirkovsky E., Macakova K., Krcmova L.K., Javorska L., Pourova J., Mercolini L., Remiao F., Novakova L., Mladenka P. (2021). Vitamin c-sources, physiological role, kinetics, deficiency, use, toxicity, and determination. Nutrients.

[15] Shivavedi N., Kumar M., Tej G., Nayak P.K. (2017). Metformin and ascorbic acid combination therapy ameliorates type 2 diabetes mellitus and comorbid depression in rats. Brain Res..

[16] Bottger F., Valles-Marti A., Cahn L., Jimenez C.R. (2021). High-dose intravenous vitamin c, a promising multi-targeting agent in the treatment of cancer. J. Exp. Clin. Cancer Res..

[17] Marwaha N. (2016). Ascorbic acid co-administration with artemisinin based combination therapies in falciparum malaria. Indian J. Med. Res..

[18] Belhachemi M.H., Boucherit-Otmani Z., Boucherit K., Belmir S. (2021). Influence of ascorbic acid and alpha-tocopherol on the autoxidation and in vitro antifungal activity of amphotericin b. Curr. Med. Mycol..

[19] Belhachemi M.H., Boucherit K., Boucherit-Otmani Z., Belmir S., Benbekhti Z. (2014). Effects of ascorbic acid and alpha-tocopherol on the therapeutic index of amphotericin b. J. Mycol. Med..

[20] Zhang X.T., Yu L.Q. (2014). Effect of vitamin c on the anti-candida albicans activity of ketoconazole. Shandong J. Anim. Sci. Vet. Med..

[21] Tsui C., Kong E.F., Jabra-Rizk M.A. (2016). Pathogenesis of candida albicans biofilm. Pathog. Dis..

[22] Zarei Mahmoudabadi A., Zarrin M., Kiasat N. (2014). Biofilm formation and susceptibility to amphotericin b and fluconazole in candida albicans. Jundishapur J. Microbiol..

[23] Chen Y., Gao Y., Li Y., Yin J. (2024). Anti-biofilm activity of assamsaponin a, theasaponin e1, and theasaponin e2 against candida albicans. Int. J. Mol. Sci..

[24] Rege A.A., Kolte D.B., Thakur D. (2024). In vitro antifungal and antibiofilm potential of ascorbic acid against candida albicans and its mixed culture with bacteria. World J. Pharm. Pharm. Sci..

[25] Danchik C., Casadevall A. (2020). Role of cell surface hydrophobicity in the pathogenesis of medically-significant fungi. Front. Cell Infect. Microbiol..

[26] Masuoka J., Hazen K.C. (1997). Cell wall protein mannosylation determines candida albicans cell surface hydrophobicity. Microbiology (Reading).

[27] El-Baz A.M., Mosbah R.A., Goda R.M., Mansour B., Sultana T., Dahms T.E.S., El-Ganiny A.M. (2021). Back to nature: Combating candida albicans biofilm, phospholipase and hemolysin using plant essential oils. Antibiotics.

[28] Ellepola A.N., Samaranayake L.P., Khan Z.U. (2016). Extracellular phospholipase production of oral candida albicans isolates from smokers, diabetics, asthmatics, denture wearers and healthy individuals following brief exposure to polyene, echinocandin and azole antimycotics. Braz. J. Microbiol..

[29] Anil S., Samaranayake L.P. (2003). Brief exposure to antimycotics reduces the extracellular phospholipase activity of candida albicans and candida tropicalis. Chemotherapy.

[30] Avci P., Freire F., Banvolgyi A., Mylonakis E., Wikonkal N.M., Hamblin M.R. (2016). Sodium ascorbate kills candida albicans in vitro via iron-catalyzed fenton reaction: Importance of oxygenation and metabolism. Future Microbiol..

[31] Guo C., Sun L., Chen X., Zhang D. (2013). Oxidative stress, mitochondrial damage and neurodegenerative diseases. Neural Regen. Res..

[32] Sakamuru S., Attene-Ramos M.S., Xia M. (2016). Mitochondrial membrane potential assay. Methods Mol. Biol..

[33] Zorova L.D., Popkov V.A., Plotnikov E.Y., Silachev D.N., Pevzner I.B., Jankauskas S.S., Babenko V.A., Zorov S.D., Balakireva A.V., Juhaszova M. (2018). Mitochondrial membrane potential. Anal. Biochem..

[34] Bonora M., Giorgi C., Pinton P. (2022). Molecular mechanisms and consequences of mitochondrial permeability transition. Nat. Rev. Mol. Cell Biol..

[35] Eleff S., Kennaway N.G., Buist N.R., Darley-Usmar V.M., Capaldi R.A., Bank W.J., Chance B. (1984). 31p nmr study of improvement in oxidative phosphorylation by vitamins k3 and c in a patient with a defect in electron transport at complex iii in skeletal muscle. Proc. Natl. Acad. Sci. USA.

[36] Komarova S.V., Ataullakhanov F.I., Globus R.K. (2000). Bioenergetics and mitochondrial transmembrane potential during differentiation of cultured osteoblasts. Am. J. Physiol. Cell Physiol..

[37] Bakalova R., Zhelev Z., Miller T., Aoki I., Higashi T. (2020). Vitamin c versus cancer: Ascorbic acid radical and impairment of mitochondrial respiration?. Oxid. Med. Cell Longev..

[38] Chandel N.S. (2021). Glycolysis. Cold Spring Harb. Perspect. Biol..

[39] Yuan W., Du Y., Yu K., Xu S., Liu M., Wang S., Yang Y., Zhang Y., Sun J. (2022). The production of pyruvate in biological technology: A critical review. Microorganisms.

[40] Dhoundiyal A., Goeschl V., Boehm S., Kubista H., Hotka M. (2022). Glycerol-3-phosphate shuttle is a backup system securing metabolic flexibility in neurons. J. Neurosci..

[41] Shi Y., Wang H., Yan Y., Cao H., Liu X., Lin F., Lu J. (2018). Glycerol-3-phosphate shuttle is involved in development and virulence in the rice blast fungus pyricularia oryzae. Front. Plant Sci..

[42] Larsson C., Pahlman I.L., Ansell R., Rigoulet M., Adler L., Gustafsson L. (1998). The importance of the glycerol 3-phosphate shuttle during aerobic growth of saccharomyces cerevisiae. Yeast.

[43] Wang Z., Zhang Q., Zhang H., Lu Y. (2024). Roles of alcohol dehydrogenase 1 in the biological activities of candida albicans. Crit. Rev. Microbiol..

[44] Gutierrez-Corona J.F., Gonzalez-Hernandez G.A., Padilla-Guerrero I.E., Olmedo-Monfil V., Martinez-Rocha A.L., Patino-Medina J.A., Meza-Carmen V., Torres-Guzman J.C. (2023). Fungal alcohol dehydrogenases: Physiological function, molecular properties, regulation of their production, and biotechnological potential. Cells.

[45] Lee S., Son H., Lee J., Min K., Choi G.J., Kim J.C., Lee Y.W. (2011). Functional analyses of two acetyl coenzyme a synthetases in the ascomycete gibberella zeae. Eukaryot. Cell.

[46] Van Den Berg M.A., Steensma H.Y. (1995). Acs2, a saccharomyces cerevisiae gene encoding acetyl-coenzyme a synthetase, essential for growth on glucose. Eur. J. Biochem..

[47] Carman A.J., Vylkova S., Lorenz M.C. (2008). Role of acetyl coenzyme a synthesis and breakdown in alternative carbon source utilization in candida albicans. Eukaryot. Cell.

[48] Lv H., Zhen C., Liu J., Yang P., Hu L., Shang P. (2019). Unraveling the potential role of glutathione in multiple forms of cell death in cancer therapy. Oxid. Med. Cell Longev..

[49] Forman H.J., Zhang H., Rinna A. (2009). Glutathione: Overview of its protective roles, measurement, and biosynthesis. Mol. Aspects Med..

[50] Lu S.C. (2013). Glutathione synthesis. Biochim. Biophys. Acta.

[51] Kitada M., Ogura Y., Monno I., Xu J., Koya D. (2021). Effect of methionine restriction on aging: Its relationship to oxidative stress. Biomedicines.

[52] Labarrere C.A., Kassab G.S. (2022). Glutathione: A samsonian life-sustaining small molecule that protects against oxidative stress, ageing and damaging inflammation. Front. Nutr..

[53] Seleem D., Chen E., Benso B., Pardi V., Murata R.M. (2016). In vitro evaluation of antifungal activity of monolaurin against candida albicans biofilms. PeerJ.

[54] Barman A., Gohain D., Bora U., Tamuli R. (2018). Phospholipases play multiple cellular roles including growth, stress tolerance, sexual development, and virulence in fungi. Microbiol. Res..

[55] Zhao Z., Zhou Y., Wang R., Xie F., Zhai Z. (2020). Expression, purification, and characterization of phospholipase b1 from candida albicans in escherichia coli. 3 Biotech.

[56] McLain N., Dolan J.W. (1997). Phospholipase d activity is required for dimorphic transition in candida albicans. Microbiology (Reading).

[57] Hube B., Hess D., Baker C.A., Schaller M., Schafer W., Dolan J.W. (2001). The role and relevance of phospholipase d1 during growth and dimorphism of candida albicans. Microbiology (Reading).

[58] Sanchez-Fresneda R., Guirao-Abad J.P., Arguelles A., Gonzalez-Parraga P., Valentin E., Arguelles J.C. (2013). Specific stress-induced storage of trehalose, glycerol and d-arabitol in response to oxidative and osmotic stress in candida albicans. Biochem. Biophys. Res. Commun..

[59] Pistoia E.S., Cosio T., Campione E., Pica F., Volpe A., Marino D., Di Francesco P., Monari C., Fontana C., Favaro M. (2022). All-trans retinoic acid effect on candida albicans growth and biofilm formation. J. Fungi.

[60] Yang B., Lei Z., Zhao Y., Ahmed S., Wang C., Zhang S., Fu S., Cao J., Qiu Y. (2017). Combination susceptibility testing of common antimicrobials in vitro and the effects of sub-mic of antimicrobials on staphylococcus aureus biofilm formation. Front. Microbiol..

[61] Possamai Rossatto F.C., Tharmalingam N., Escobar I.E., d’Azevedo P.A., Zimmer K.R., Mylonakis E. (2021). Antifungal activity of the phenolic compounds ellagic acid (ea) and caffeic acid phenethyl ester (cape) against drug-resistant candida auris. J. Fungi.

